# Correction: Exploring the non-communicable disease (NCD) network of multi-morbid individuals in India: A network analysis

**DOI:** 10.1371/journal.pgph.0001809

**Published:** 2023-04-04

**Authors:** The PLOS Global Public Health Staff

## Notice of Republication

This article was republished on December 7, 2022 to replace the incorrect editor (“PLOS Manuscript Reassignment”) with the correct editor (Prabhdeep Kaur). The publisher apologizes for the errors. Please download this article again to view the correct version. The originally published, uncorrected article and the republished, corrected articles are provided here for reference.

## Supporting information

S1 File Originally published, uncorrected article. (PDF)

S2 File Republished, corrected article. (PDF)
