# Supplementary material for: Exploring the non-communicable disease (NCD) network of multi-morbid individuals in India: A network analysis
Source: PLOS Glob Public Health. 2022 Jun 30;2(6):e0000512. doi: 10.1371/journal.pgph.0000512 (PMC10021153; doi:10.1371/journal.pgph.0000512)

Supporting Document S1 Fig

S1 Fig. Full multimorbidity networks for (A) women and (B) men aged 45 years and above for sixteen non-communicable diseases, LASI, 2017-18

A.

B.

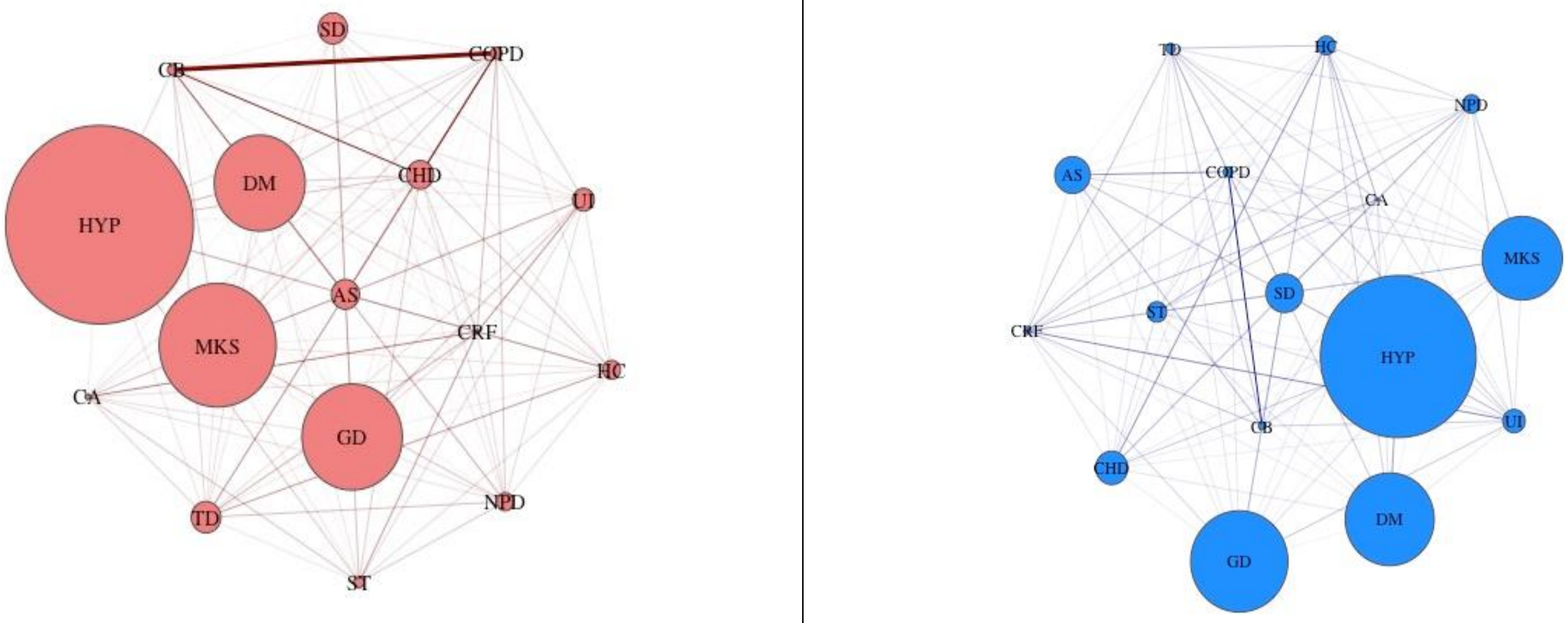

Supplement: S1 Fig — (PDF) [file pgph.0000512.s001.pdf]
