# Supplementary material for: Exploring the non-communicable disease (NCD) network of multi-morbid individuals in India: A network analysis
Source: PLOS Glob Public Health. 2022 Jun 30;2(6):e0000512. doi: 10.1371/journal.pgph.0000512 (PMC10021153; doi:10.1371/journal.pgph.0000512)
Supplement: S3 Table — (PDF) [file pgph.0000512.s004.pdf]

Supporting Document: S3 Table

S3 Table. Age-adjusted association between selected non-communicable diseases (NCDs) segregated by sex, among older adults in India, LASI, 2017-18.

Non-communicable Disease 1 (X)	Non-communicable Disease 2 (Y)	Age-adjusted Odds Ratio (p-value)	
		Men	Women
Asthma (AS)	Musculoskeletal Disorder (MKS)	1.19 (0.000)	0.67 (0.000)
Asthma (AS)	Cancer (CA)	0.44 (0.000)	0.54 (0.000)
Asthma (AS)	Chronic Bronchitis (CB)	1.84 (0.000)	2.90 (0.000)
Asthma (AS)	Chronic Renal Failure (CRF)	0.71(0.000)	0.87 (0.000)
Asthma (AS)	Coronary Obstructive Pulmonary Disorder (COPD)	3.08 (0.000)	2.35 (0.000)
Asthma (AS)	Diabetes Miletus (DM)	0.29 (0.000)	0.58 (0.000)
Asthma (AS)	Gastrointestinal Disorder (GD)	0.67 (0.000)	0.59 (0.000)
Asthma (AS)	Chronic Heart Disease (CHD)	0.62 (0.000)	0.86 (0.000)
Asthma (AS)	High Cholesterol (HC)	0.36 (0.000)	0.73 (0.000)
Asthma (AS)	Hypertension (HYP)	0.32(0.000)	0.48 (0.000)
Asthma (AS)	Urinary Incontinence (UI)	0.89 (0.000)	0.94 (0.000)
Asthma (AS)	Neurological and Psychiatric Disorder (NPD)	0.58 (0.000)	1.17 (0.000)
Asthma (AS)	Skin Diseases (SD)	0.62 (0.000)	1.01(0.000)
Asthma (AS)	Stroke (ST)	0.36 (0.000)	0.68 (0.000)
Asthma (AS)	Thyroid Disease (THY)	0.30 (0.000)	1.17 (0.000)
Musculoskeletal Disorder (MKS)	Cancer (CA)	0.72 (0.000)	0.37 (0.000)
Musculoskeletal Disorder (MKS)	Chronic Bronchitis (CB)	1.33 (0.000)	1.42 (0.000)
Musculoskeletal Disorder (MKS)	Chronic Renal Failure (CRF)	1.00 (0.000)	0.59 (0.000)
Musculoskeletal Disorder (MKS)	Coronary Obstructive Pulmonary Disorder (COPD)	0.91 (0.000)	1.46 (0.000)
Musculoskeletal Disorder (MKS)	Diabetes Miletus (DM)	0.39 (0.000)	0.46 (0.000)
Musculoskeletal Disorder (MKS)	Gastrointestinal Disorder (GD)	0.92 (0.000)	0.69 (0.000)
Musculoskeletal Disorder (MKS)	Chronic Heart Disease (CHD)	0.61(0.000)	0.91 (0.000)
Musculoskeletal Disorder (MKS)	High Cholesterol (HC)	0.62 (0.000)	0.82 (0.000)
Musculoskeletal Disorder (MKS)	Hypertension (HYP)	0.31 (0.000)	0.49 (0.000)
Musculoskeletal Disorder (MKS)	Urinary Incontinence (UI)	0.89 (0.000)	0.85 (0.000)
Musculoskeletal Disorder (MKS)	Neurological and Psychiatric Disorder (NPD)	1.47 (0.000)	1.56 (0.000)
Musculoskeletal Disorder (MKS)	Skin Diseases (SD)	0.7 (0.000)	0.73 (0.000)
Musculoskeletal Disorder (MKS)	Stroke (ST)	0.7 (0.000)	0.68 (0.000)
Musculoskeletal Disorder (MKS)	Thyroid Disease (THY)	0.77 (0.000)	0.73 (0.000)
Cancer (CA)	Chronic Bronchitis (CB)	0.44 (0.000)	0.05 (0.000)
Cancer (CA)	Chronic Renal Failure (CRF)	1.79 (0.000)	3.33 (0.000)
Cancer (CA)	Coronary Obstructive Pulmonary Disorder (COPD)	0.83 (0.000)	0.85 (0.000)
Cancer (CA)	Diabetes Miletus (DM)	0.98 (0.000)	0.43 (0.000)
Cancer (CA)	Gastrointestinal Disorder (GD)	0.56 (0.000)	0.93 (0.000)
Cancer (CA)	Chronic Heart Disease (CHD)	0.93 (0.000)	0.65 (0.000)
Cancer (CA)	High Cholesterol (HC)	2.25 (0.000)	0.95 (0.000)
Cancer (CA)	Hypertension (HYP)	0.58 (0.000)	0.55(0.000)
Cancer (CA)	Urinary Incontinence (UI)	0.96 (0.000)	1.06 (0.000)
Cancer (CA)	Neurological and Psychiatric Disorder (NPD)	0.93 (0.000)	0.90 (1.000)
Cancer (CA)	Skin Diseases (SD)	0.75 (0.000)	0.23(0.000)
Cancer (CA)	Stroke (ST)	1.21 (0.000)	1.62 (0.000)
Cancer (CA)	Thyroid Disease (THY)	1.45 (0.000)	1.25 (0.000)
Chronic Bronchitis (CB)	Chronic Renal Failure (CRF)	1.71 (0.000)	0.24 (0.000)
Chronic Bronchitis (CB)	Coronary Obstructive Pulmonary Disorder (COPD)	5.94 (0.000)	17.27 (0.000)
Chronic Bronchitis (CB)	Diabetes Miletus (DM)	0.45 (0.000)	1.72 (0.000)
Chronic Bronchitis (CB)	Gastrointestinal Disorder (GD)	0.85 (0.000)	0.46 (0.000)
Chronic Bronchitis (CB)	Chronic Heart Disease (CHD)	0.56 (0.000)	5.37 (0.000)
Chronic Bronchitis (CB)	High Cholesterol (HC)	0.74 (0.000)	0.58 (0.000)
Chronic Bronchitis (CB)	Hypertension (HYP)	0.39 (0.000)	1.21 (0.000)
Chronic Bronchitis (CB)	Urinary Incontinence (UI)	1.52 (0.000)	0.84 (0.000)
Chronic Bronchitis (CB)	Neurological and Psychiatric Disorder (NPD)	1.06 (0.000)	0.75 (0.000)
Chronic Bronchitis (CB)	Skin Diseases (SD)	0.75 (0.000)	0.41 (0.000)
Chronic Bronchitis (CB)	Stroke (ST)	0.24 (0.000)	0.28 (0.000)
Chronic Bronchitis (CB)	Thyroid Disease (THY)	1.71 (0.000)	1.14 (0.000)
Chronic Renal Failure (CRF)	Coronary Obstructive Pulmonary Disorder (COPD)	1.90 (0.000)	1.91 (0.000)
Chronic Renal Failure (CRF)	Diabetes Miletus (DM)	0.59 (0.000)	0.56 (0.000)
Chronic Renal Failure (CRF)	Gastrointestinal Disorder (GD)	1.17 (0.000)	1.07 (0.000)
Chronic Renal Failure (CRF)	Chronic Heart Disease (CHD)	0.76 (0.000)	1.23 (0.000)

Chronic Renal Failure (CRF)	High Cholesterol (HC)	0.73 (0.000)	1.51 (0.000)
Chronic Renal Failure (CRF)	Hypertension (HYP)	0.46 (0.000)	0.89 (0.000)
Chronic Renal Failure (CRF)	Urinary Incontinence (UI)	3.28 (0.000)	2.82 (0.000)
Chronic Renal Failure (CRF)	Neurological and Psychiatric Disorder (NPD)	1.05(0.040)	1.01 (0.0027)
Chronic Renal Failure (CRF)	Skin Diseases (SD)	0.72 (0.000)	0.85 (0.000)
Chronic Renal Failure (CRF)	Stroke (ST)	0.91 (0.000)	2.61 (0.000)
Chronic Renal Failure (CRF)	Thyroid Disease (THY)	1.65 (0.000)	2.08 (0.000)
Coronary Obstructive Pulmonary Disorder (COPD)	Diabetes Miletus (DM)	0.38 (0.000)	1.48 (0.000)
Coronary Obstructive Pulmonary Disorder (COPD)	Gastrointestinal Disorder (GD)	0.71(0.000)	0.37 (0.000)
Coronary Obstructive Pulmonary Disorder (COPD)	Chronic Heart Disease (CHD)	0.63(0.000)	5.31 (0.000)
Coronary Obstructive Pulmonary Disorder (COPD)	High Cholesterol (HC)	0.42(0.000)	0.83(0.000)
Coronary Obstructive Pulmonary Disorder (COPD)	Hypertension (HYP)	0.35(0.000)	1.07(0.000)
Coronary Obstructive Pulmonary Disorder (COPD)	Urinary Incontinence (UI)	1.00 (0.000)	1.37(0.000)
Coronary Obstructive Pulmonary Disorder (COPD)	Neurological and Psychiatric Disorder (NPD)	0.49 (0.000)	1.56(0.000)
Coronary Obstructive Pulmonary Disorder (COPD)	Skin Diseases (SD)	0.58 (0.000)	0.65(0.000)
Coronary Obstructive Pulmonary Disorder (COPD)	Stroke (ST)	0.67 (0.000)	0.25(0.000)
Coronary Obstructive Pulmonary Disorder (COPD)	Thyroid Disease (THY)	0.96 (0.000)	1.29(0.000)
Diabetes Miletus (DM)	Gastrointestinal Disorder (GD)	0.35 (0.000)	0.28(0.000)
Diabetes Miletus (DM)	Chronic Heart Disease (CHD)	0.78 (0.000)	1.53(0.000)
Diabetes Miletus (DM)	High Cholesterol (HC)	1.38 (0.000)	1.11(0.000)
Diabetes Miletus (DM)	Hypertension (HYP)	2.77 (0.000)	1.91(0.000)
Diabetes Miletus (DM)	Urinary Incontinence (UI)	0.61 (0.000)	0.62(0.000)
Diabetes Miletus (DM)	Neurological and Psychiatric Disorder (NPD)	0.43 (0.000)	0.44(0.000)
Diabetes Miletus (DM)	Skin Diseases (SD)	0.63 (0.000)	0.34(0.000)
Diabetes Miletus (DM)	Stroke (ST)	0.81 (0.000)	0.81(0.000)
Diabetes Miletus (DM)	Thyroid Disease (THY)	0.57 (0.000)	0.93(0.000)
Gastrointestinal Disorder (GD)	Chronic Heart Disease (CHD)	0.52 (0.000)	0.54(0.000)
Gastrointestinal Disorder (GD)	High Cholesterol (HC)	0.66 (0.000)	0.53(0.000)
Gastrointestinal Disorder (GD)	Hypertension (HYP)	0.37 (0.000)	0.42(0.000)
Gastrointestinal Disorder (GD)	Urinary Incontinence (UI)	1.53 (0.000)	1.70 (0.000)
Gastrointestinal Disorder (GD)	Neurological and Psychiatric Disorder (NPD)	0.67 (0.000)	0.86(0.000)
Gastrointestinal Disorder (GD)	Skin Diseases (SD)	1.03 (0.000)	1.31(0.000)
Gastrointestinal Disorder (GD)	Stroke (ST)	0.62 (0.000)	0.55(0.000)
Gastrointestinal Disorder (GD)	Thyroid Disease (THY)	0.78 (0.000)	0.68(0.000)
Chronic Heart Disease (CHD)	High Cholesterol (HC)	2.99 (0.000)	1.59(0.000)
Chronic Heart Disease (CHD)	Hypertension (HYP)	1.53 (0.000)	1.72(0.000)
Chronic Heart Disease (CHD)	Urinary Incontinence (UI)	0.53(0.000)	0.75(0.000)
Chronic Heart Disease (CHD)	Neurological and Psychiatric Disorder (NPD)	0.89 (0.000)	0.95(0.000)
Chronic Heart Disease (CHD)	Skin Diseases (SD)	0.38 (0.000)	0.35 (0.000)
Chronic Heart Disease (CHD)	Stroke (ST)	1.09 (0.000)	1.41 (0.000)
Chronic Heart Disease (CHD)	Thyroid Disease (THY)	0.73 (0.000)	0.78 (0.000)
High Cholesterol (HC)	Hypertension (HYP)	1.60 (0.000)	1.33 (0.000)
High Cholesterol (HC)	Urinary Incontinence (UI)	0.73 (0.000)	0.94 (0.000)
High Cholesterol (HC)	Neurological and Psychiatric Disorder (NPD)	1.16 (0.000)	0.93 (0.000)
High Cholesterol (HC)	Skin Diseases (SD)	0.57 (0.000)	0.65 (0.000)
High Cholesterol (HC)	Stroke (ST)	0.85 (0.147)	1.00 (0.155)
High Cholesterol (HC)	Thyroid Disease (THY)	1.89 (0.000)	2.63 (0.000)
Hypertension (HYP)	Urinary Incontinence (UI)	0.38(0.000)	0.4 (0.000)
Hypertension (HYP)	Neurological and Psychiatric Disorder (NPD)	0.61(0.000)	0.38(0.000)
Hypertension (HYP)	Skin Diseases (SD)	0.47 (0.000)	0.32 (0.000)
Hypertension (HYP)	Stroke (ST)	0.40 (0.000)	1.48 (0.000)
Hypertension (HYP)	Thyroid Disease (THY)	0.79 (0.000)	0.79 (0.000)
Urinary Incontinence (UI)	Neurological and Psychiatric Disorder (NPD)	1.26 (0.000)	1.05(0.000)
Urinary Incontinence (UI)	Skin Diseases (SD)	0.89 (0.000)	0.89 (0.000)
Urinary Incontinence (UI)	Stroke (ST)	1.05 (0.000)	1.06 (0.000)
Urinary Incontinence (UI)	Thyroid Disease (THY)	1.51 (0.000)	0.92 (0.000)
Neurological and Psychiatric Disorder (NPD)	Skin Diseases (SD)	0.51 (0.000)	0.67 (0.000)
Neurological and Psychiatric Disorder (NPD)	Stroke (ST)	1.95 (0.000)	1.49(0.000)
Neurological and Psychiatric Disorder (NPD)	Thyroid Disease (THY)	1.02 (0.000)	1.79 (0.000)
Skin Disease (SD)	Stroke (ST)	0.50 (0.000)	0.69 (0.000)
Skin Disease (SD)	Thyroid Disease (THY)	1.12 (0.000)	0.71 (0.000)
Stroke (ST)	Thyroid Disease (THY)	0.67 (0.000)	0.67(0.000)
