# Supplementary material for: Exploring the non-communicable disease (NCD) network of multi-morbid individuals in India: A network analysis
Source: PLOS Glob Public Health. 2022 Jun 30;2(6):e0000512. doi: 10.1371/journal.pgph.0000512 (PMC10021153; doi:10.1371/journal.pgph.0000512)
Supplement: Attachment — Submitted filename: Comments.docx [file pgph.0000512.s005.docx]

**Editorial Comments and Journal Requirements**

**Comment #1**: Please provide an Author Summary. This should appear in your manuscript between the Abstract (if applicable) and the Introduction, and should be 150–200 words long. The aim should be to make your findings accessible to a wide audience that includes both scientists and non-scientists. Sample summaries can be found on our website under Submission Guidelines: <https://journals.plos.org/globalpublichealth/s/submission-guidelines#loc-parts-of-a-submission>
**Reply #1:** Thank you for the observation. We have added the Author Summary as suggested. Please refer to the revised submission.


**Comment #2:** We note that you have indicated that data from this study are available upon request. PLOS only allows data to be available upon request if there are legal or ethical restrictions on sharing data publicly. For information on unacceptable data access restrictions, please see <http://journals.plos.org/plosone/s/data-availability#loc-unacceptable-data-access-restrictions>. Before we proceed with your manuscript, please address the following prompts:

A. Does your manuscript contain third-party data?

B. If so, please confirm that the authors did not have any special access privileges that others would not have.

C. Please also provide all relevant data set names, variables, instructions, etc. that an independent researcher would need in order to access or request your minimal data set from the third-party data owner.

We will update your Data Availability statement upon your response.

**Reply #2:** The data used in the study is taken from the Longitudinal Ageing Study in India (LASI), wave-1, 2019-21. We have not received any special access privileges.

The data has been archived in the public repository of the Longitudinal Ageing Study in India, 2017-18. The data can be accessed using the link: <https://www.iipsindia.ac.in/content/lasi-wave-i>. Access to the dataset requires registration and is granted only for legitimate research purposes. The application to access dataset is available at <https://iipsindia.ac.in/sites/default/files/LASI_DataRequestForm_0.pdf>. A duly-filled form has to be emailed to Information, Communication and Technology (ICT) Unit held at the International Institute for Population Sciences, Mumbai at [datacenter@iipsindia.ac.in](mailto:datacenter@iipsindia.ac.in).

**Author’s reply on the Journal Requirements and comments received**

We would like to thank all the reviewers for their thoughtful comments and efforts towards improving our manuscript. The suggestions offered have been extremely helpful in revising the manuscript. We have tried to incorporate all the changes to the best of our ability. Please find the response to all the comments highlighted in a point-by-point basis. We hope the revised manuscript is appropriate for publication in the journal. But we are open to further revisions if required.

**Editorial Comments and Journal Requirements**

**Comment #1:** Please amend your Financial Disclosure statement. If you did not receive any funding for this study, please simply state: “The authors received no specific funding for this work.”
**Reply #1:** Statement on financial disclosure has been added in the revised submission.

**Comment #2:** Please update your Competing Interests statement. If you have no competing interests to declare, please state: “The authors have declared that no competing interests exist.”
**Reply #2:** Statement on competing interest has been added in the revised submission.

**Comment #3:** Please note that your Data Availability Statement is currently missing a direct link to access each database. If your manuscript is accepted for publication, you will be asked to provide these details on a very short timeline. We therefore suggest that you provide this information now, though we will not hold up the peer review process if you are unable.
**Reply #3:** Statement on data availability has been amended as per the suggestion.

**Comment #4:** Please provide separate figure files in .tif or .eps format only and ensure that all files are under our size limit of 20MB. For more information about how to convert your figure files please see our guidelines: <https://journals.plos.org/globalpublichealth/s/figures>
**Reply #4:** Separate figure files in the required format have been submitted as suggested.

**Comment #5:** Please include a copy of Table 1 which you refer to in your text on page 8.
**Reply #5:** We have added a copy of Table 1 in the revised text as suggested. Please refer to page number [xxx] of the revised text.

**Reviewers' comments**

**Reviewer #1:** Palash Chandra Banik

**Comment #6:** It is very interesting and important one on Noncommunicable Diseases. Specially the authors try to analysed the multi-morbidity of NCDs among the patients. Particularly, multi-morbidity always makes the disease progression more complex and quality of life become poor. To prevention of multi-morbidity, we need to know first about the pattern of it. 
**Reply #6:** Thank you for the kind appreciation on our work. There are no comments to be addressed here.

**Reviewer #2:** Rajat Das Gupta

Puri and colleagues aimed to decode the complexities of multiple non-communicable diseases
among older adults (individuals aged 45 years or older) in India using network analysis. The manuscript has merit and definitely important considering the novel analysis techniques which the authors used. However, authors should address the following comments in order to improve the quality of the manuscript.

**Comment #7:** Please provide description of the data source and the geographic area it covered.
**Reply #7:** Thank you for the suggestion. We have added a description of the data source and its geographic area as suggested by the reviewer. Please refer to page 4-6 of the revised text.

**Comment #8:** Data collection process including sampling, sampling frame, tools and questionnaires should be described.
**Reply #8:** Thank you for the suggestion. We have added a description on sampling, sampling frame, tools and questionnaire as suggested. Please refer to page 4-6 of the revised text.

**Comment #9:** The first and foremost limitations of the study is the multimorbidity’s were self-reported. A description regarding the data collection process and how the participants were asked about the multimorbidity should be added.
**Reply #9:** Thank you for the observation. We realise that the use of self-reported data might add some bias, however, this is the only feasible source available at national-level. Given this, we have added a description on the data collection process as suggested. Please refer to page 6 of the revised text.

**Comment #10:** Explicit explanation of the following information needed in the methods section:
• Number of nodes
• Number of edges
• Diameter
• Density
What is the significance of the number of these elements? If the number is higher, what does it imply? If the number is lower, what does it imply? Similarly, what is the implication of Degree, Closeness Centrality (in %), Betweenness Centrality (in %)?
**Reply #10**: Thank you for the observation. We have added the aforementioned information in the methods section as suggested. Similarly, information on the implications of these information has also been added in the revised text. Please refer to page 8-9 of the revised text.

**Comment #11:** In the first line of discussion section, the authors mentioned that it is a nationally representative sample. Did the authors adjust the weighting and cluster effect during analysis?
**Reply #11:** Thank you for the suggestion. All the estimates provided and visualized in the study are adjusted for weighting and clustering effect. We have added the same, please refer to page 9 and line 196-197 of the revised text.

**Comment #12:** Please highlight policy level implications of the research.
**Reply #12:** Thank you for the suggestion. We have added policy level implications of the research as suggested. Please refer to page 17 and line 338-344 of the revised text.
